# Cardiac Arrest in a Vitamin D–Deficient Infant

**DOI:** 10.1177/2333794X18765064

**Published:** 2018-03-20

**Authors:** Ana L. Creo, Peter J. Tebben, Philip R. Fischer, Thomas D. Thacher, Siobhan T. Pittock

**Affiliations:** 1 Mayo Clinic, Rochester, MN, USA

## Introduction

Vitamin D deficiency remains a significant pediatric global health problem affecting children in both high- and low-income countries; new international consensus recommendations to prevent rickets target 400 IU/day of vitamin D in infants until 12 months of age.^1,2^ Despite widespread supplementation recommendations, vitamin D deficiency is highly prevalent among infants with additional risk factors including those who are immigrants, exclusively breastfed, born to vitamin D–deficient mothers, have darker skin pigmentation, and live at high latitudes.

While children with vitamin D deficiency are at risk for developing rickets, rarely, they may have more life-threatening consequences. Severe sequelae of vitamin D deficiency include dilated cardiomyopathy and hypocalcemic seizures, but cardiopulmonary arrest is a rare presentation. Here we report a case of an infant with vitamin D deficiency resulting in cardiopulmonary arrest and severe neurologic sequelae.

## Case Presentation

A 7-month-old exclusively breastfed, firstborn infant to black East African immigrant parents was brought to our emergency department (ED) in March after experiencing an at-home, witnessed cardiopulmonary arrest. She was born in Rochester, Minnesota, at term via uncomplicated pregnancy and vaginal delivery in late summer. She did well until 4 months of age when she had slower weight gain and intermittent noisy breathing that prompted several visits to the ED. At 6 months of age, she presented to the ED after 2 spells that included “stiffening” and “not responding.” An ENT surgeon performed a bedside laryngoscopy and did not note evidence of obstruction. She followed-up in primary care and continued to have slow weight gain, but no laboratory testing was performed. She commenced mefloquine in preparation for an upcoming trip to Africa.

**Figure 1.**
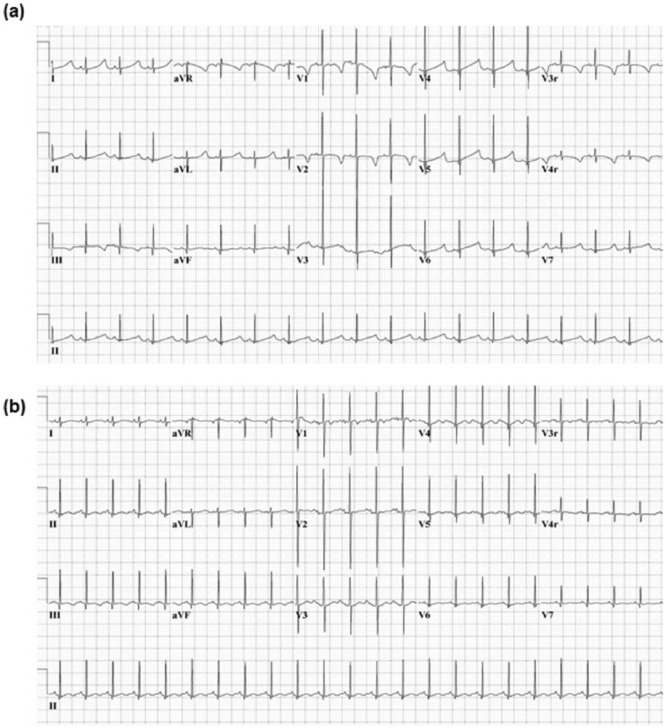
(A) Initial electrocardiogram (ECG) demonstrating prolonged QTc 535 ms, total calcium 4.8 mg/dL (1.2 mmol/L). (B) The ECG after correction of hypocalcemia with a normalized QTc 351 ms, total calcium 9.6 mg/dL (2.4 mmol/L).

At 7 months of age, the infant awoke from a nap, had noisy breathing, and started crying. She “went tense” in the mother’s arms, becoming apneic and pulseless. Cardiopulmonary resuscitation was immediately initiated by the family and continued by emergency first responders. Spontaneous circulation returned after a second dose of epinephrine, 24 minutes after initial arrest. Initial laboratory tests revealed severe acidosis with a pH <6.8 (normal = 7.35-7.45), lactate of 15.3 mmol/L (normal = 0.6-3.2 mmol/L), pCO_2_ of 50 mm Hg, normal blood glucose, and hypocalcemia with ionized and total calcium values of 2.7 mg/dL ([0.7 mmol/L], normal = 3.7-5.9 mg/dL) and 4.8 mg/dL ([1.2 mmol/L], normal = 9-11 mg/dL), respectively. She required a high-dose intravenous calcium infusion (10-12 mg/kg/h of calcium chloride) for 4 days postarrest. Initial electrocardiography (ECG) showed a prolonged QTc interval of 535 ms, which resolved with correction of hypocalcemia (Figure 1).

Other laboratory tests showed an appropriately elevated parathyroid hormone of 305 pg/mL ([305 ng/L], normal = 15-65 pg/mL), low urinary calcium excretion of <1 mg/dL, and spot urine calcium: creatinine of <0.03 mg/dL (Table 1). 25-Hydroxyvitamin D was unmeasurable with an elevated alkaline phosphatase of 723 U/L ([12.3 Ukat/L], normal = 150-420 U/L). After full endocrine, metabolic, cardiac, infectious disease, and neurologic investigations and consultations, no cause aside from hypocalcemia was found for her arrest. Her head imaging showed evidence of severe hypoxic ischemic injury. ECG and cardiac examination were entirely normal after correction of hypocalcemia. Chest imaging was normal with no evidence of cardiomegaly. Due to temporal association of mefloquine initiation and the arrest, pharmacogenomic testing was performed. She was an extensive (normal) CYP3A4 metabolizer, the only CYP pathway considered relevant to mefloquine metabolism.

**Table 1.** Initial Laboratory Studies.

	Value	Normal^a^		Value	Normal^a^
*CBC*			*Immunology/microbiology*		
Hemoglobin	10.3 g/dL	10.5-13.5 g/dL	CRP	<3.0 mg/L	<3.0 mg/dL
Hematocrit	33.5%	33.0% to 40.0%	Respiratory virus panel	Negative	
Leukocytes	19.0 × 10^9^/L	6.0-11.0 × 10^9^/L	Influenza and RSV	Negative	
Platelet count	294 × 10^9^/L	150-450 × 10^9^/L			
			*Endocrine*		
*Blood chemistry*			Parathyroid hormone	305 pg/mL	15-65 pg/mL
Sodium	141 mEq/L	130-145 mEq/L	25-Hydroxyvitamin D	<6 ng/dL	20-50 ng/mL
Potassium	3.1 mEq/L	4.1-5.3 mEq/L	1,25-Dihydroxyvitamin D	13 pg/mL	16-65 pg/mL
Glucose	318 mg/dL	60-100 mg/dL			
Chloride	100 mEq/L	97-106 mEq/L	*Metabolic*		
Bicarbonate	5 mEq/L	19-24 mEq/L	Amino acids	Normal	
Creatinine	0.2 mg/dL	0.2-0.4 mg/dL	Acylcarnitines	Normal	
Blood urea nitrogen	7 mg/dL	5-18 mg/dL	Urine amino acids	Normal	
Anion gap	32		Organic acids	Normal	
Total calcium	4.8 mg/dL	9-11 mg/dL			
Ionized calcium	2.7 mg/dL	3.7-5.9 mg/dL	*Arterial blood gas*		
pH	<6.8		PCO_2_	50 mm Hg	32-48 mm Hg
Phosphorous	5.0 mg/dL	4-6.5 mg/dL	pH	<6.8	7.35-7.45
Magnesium	2.0 mg/dL	1.6-2.4 mg/dL	Base excess	<−25 mmol/L	
Lactate	15.2 mmol/L	0.6-3.2 mmol/L			
AST	30 U/L	9-80 U/L			
Bilirubin, total/direct	0.2/<0.1 mg/dL	<1.5/<0.2 mg/dL			
Alkaline phosphatase	723 U/L	150-420 U/L			

Abbreviations: CBC, complete blood count; AST, aspartate aminotransferase; CRP, C-reactive protein test; RSV, respiratory syncytial virus.

a Definitions based on *The Harriet Lane Handbook*, 21st edition.^12^

**Figure 2.**
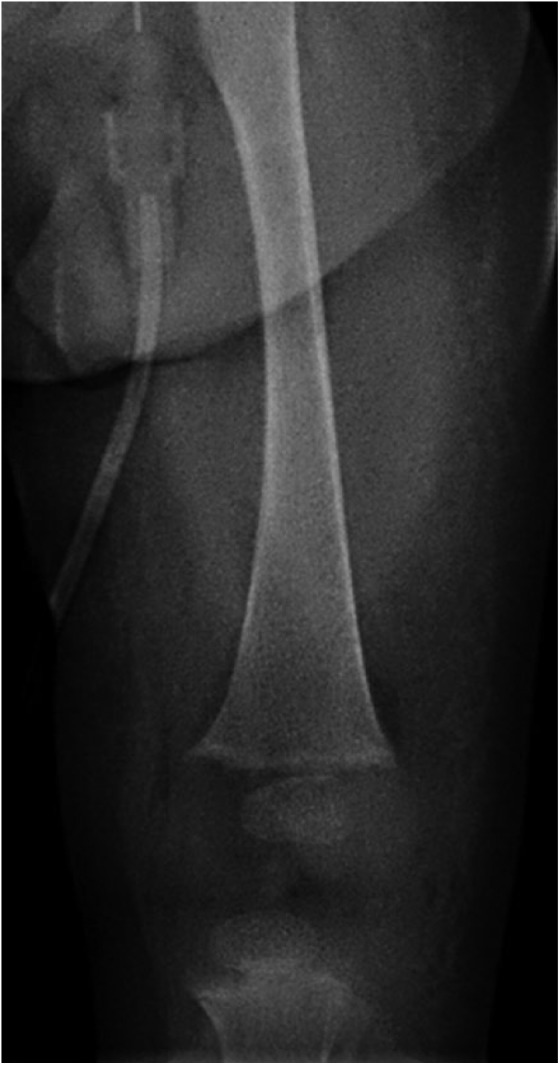
Mild to moderate rickets of the distal femur.

Additional history revealed that she had been breastfed since birth without any formula or vitamin D supplementation. The family had begun introducing solids, mainly rice and fruits, within the past month. The infant had been prescribed vitamin D drops, but the family never initiated supplementation. The infant’s mother also had a low 25-hydroxyvitamin D concentration of 10 ng/mL ([25 nmol/L], normal = 20-50 ng/mL optimal, <10 severe deficiency). Radiographic imaging showed moderate rickets with characteristic metaphyseal fraying and slight cupping (Figure 2). The infant was given 2000 IU of vitamin D and 0.25 µg of calcitriol daily and continued oral calcium (50 mg/kg/day elemental calcium). Her hypocalcemia resolved, and calcitriol and supplemental calcium were discontinued 2 weeks after initial presentation; vitamin D supplementation was continued. Unfortunately, she suffered sequelae of severe hypoxic ischemic injury with minimal neurological recovery to date.

## Discussion

The American Academy of Pediatrics recommends vitamin D supplementation of all breastfed infants. However, compliance with the recommendation remains poor, and not all breastfed children carry the same risk of vitamin D deficiency.^3^

Despite the number of children with rickets across the globe, cardiorespiratory arrest secondary to severe hypocalcemia is not frequently recognized or reported. Dilated cardiomyopathy has been considered the most serious consequence of vitamin D deficiency rickets and is well described. All cases described are in children younger than 2 years of age, with the largest case series of 61 infants citing 5 months of age as the mean age at presentation.^4^ Another series of 16 English infants who acquired cardiac failure secondary to vitamin D deficiency found all 16 had darker skin pigmentation, almost all were exclusively breastfed, and most were diagnosed in winter.^5^ The average total calcium concentration at diagnosis was 6 mg/dL (1.5 mmol/L) with a range of 4.3 to 6.9 mg/dL (1.1-1.8 mmol/L); average ionized calcium was 2.8 mg/dL (0.7 mmol/L) with a range of 1.6 to 4.1 mg/dL (0.4-1.0 mmol/L). Of the 16 babies, 6 had cardiac arrest at presentation; all had clinical evidence of heart failure due to dilated cardiomyopathy. The exact mechanism for cardiomyopathy progression remains unknown. Severe hypocalcemia likely is contributory with calcium playing a critical role in ventricular contractility. Interestingly, vitamin D deficiency may independently contribute to heart failure progression.^6,7^ Our patient had no evidence of cardiomyopathy, and to our knowledge only 2 other cases of cardiac arrest without dilated cardiomyopathy have been described.^8,9^

Vitamin D deficiency leading to hypocalcemic stridor is an infrequent presentation of rickets. Other children with rickets have developed stridor and laryngospasm.^8,10.11^ Crying and hyperventilation, as occurred in our patient, could lead to alkalosis, precipitating further reduction in ionized calcium with subsequent laryngospasm, tetany, and cardiac arrest.

It remains unclear if mefloquine had any role in our patient’s arrest. She initiated mefloquine 2 weeks prior to her arrest, though it is thought that mefloquine causes less QT prolongation compared with other antimalarial drugs. Her normal CYP3A4 activity, known to be responsible for mefloquine metabolism, and the normalization of QTc after correction of hypocalcemia, argues against any role for mefloquine in her arrest. However, an additive effect of mefloquine to the hypocalcemia-induced prolonged QTc cannot be completely ruled out.

## Conclusion

Children with vitamin D deficiency are at risk not only for rickets but also more severe, life-threatening complications. This case highlights the critical importance of adequate vitamin D supplementation in infancy. This is particularly true for children at highest risk, including infants with dark skin pigmentation, born in the fall/winter months, living at northern latitude, and infants of recent immigrants and of mothers with vitamin D deficiency. With the incidence of nutritional rickets on the rise, hypocalcemia and vitamin D deficiency need to be recognized as potential causes of heart failure, seizures, stridor, apnea, and cardiac arrest in infancy. These consequences are entirely preventable with routine infant vitamin D supplementation.
